# Differences in Behavior between Normal and Atopic Keratinocytes in Culture: Pilot Studies

**DOI:** 10.3390/vetsci9070329

**Published:** 2022-06-30

**Authors:** Rosanna Marsella, Kim Ahrens, Rachel Wilkes

**Affiliations:** Department of Small Animal Clinical Sciences, College of Veterinary Medicine, University of Florida, Gainesville, FL 32611, USA; sciencematters2me@gmail.com (K.A.); rachelsusansanford@gmail.com (R.W.)

**Keywords:** atopic dermatitis, dogs, keratinocytes, TEER, Tight junction, filaggrin

## Abstract

Skin barrier dysfunction is important in atopic dermatitis and can be secondary to inflammation. Observation of keratinocytes in culture may show intrinsic differences. TransEpithelial Electrical Resistance (TEER) measures epithelial permeability. We cultured normal and atopic keratinocytes and found that TEER of atopic keratinocytes was significantly lower (*p* < 0.0001) than that of normals. Atopic keratinocytes grew upwards, first creating isolated dome-like structures and later horizontally into a monolayer. At time of confluence (D0), atopic keratinocytes were more differentiated, with higher filaggrin gene expression than normals. No differences existed between groups for TJ proteins (claudin, occludin, and Zonula Occludens-1) on D0 and D6. On D6, claudin and occludin were higher than D0, in normal (*p* = 0.0296 and *p* = 0.0011) and atopic keratinocytes (*p* = 0.0348 and 0.0491). Immunofluorescent staining showed nuclear location of filaggrin on D0 and cytoplasmic on D6. ANOVA showed increased cell size from D0 to D6 in both groups (effect of time, *p* = 0.0076) but no differences between groups. Significant subject effect (*p* = 0.0022) was found, indicating that cell size was subject-dependent but not disease-dependent. No difference for continuity for TJ protein existed between groups. These observations suggest that decreased TEER in atopics is not linked to TJ differences but is possibly linked to different growth behavior.

## 1. Introduction

Proper keratinocyte differentiation and connection are important factors for effective skin barrier function, for the control of water loss, and for absorption of external substances. Skin barrier dysfunction and abnormalities in keratinocyte differentiation appear to play an important role in canine atopic dermatitis (AD) [1,2,3]. Debate exists as to whether some of these changes are primary and linked to genetic mutations [4,5] or whether they are mainly observed due to inflammation [6,7]. 

Tight junctions (TJ) are important for the connection between epithelial cells and the regulation of epithelial permeability [8,9]. Several proteins have been described within the TJ complex such as occludin, claudin, and Zonula Occludens-1 (ZO-1) [10]. The expression of TJ proteins is modulated by inflammation and decreased by T helper 2 (Th2) cytokines [11,12,13,14]. Decreased immunostaining of TJ proteins has been reported in biopsies performed on non-lesional skin of atopic dogs compared to normal controls [15,16]. Since non-lesional skin in atopics still has a level of low-grade inflammation and is not the same as normal skin, it is possible that some of the changes in TJ expression may actually still be secondary to inflammation even in clinically non-lesional skin. 

Filaggrin is crucial for barrier function and alterations of filaggrin can affect TJ expression [17] as well as have an impact on cell morphology and distribution of proteins important for intercellular connection [18]. The nuclear translocation of filaggrin is considered important for nuclear dissolution in the process of keratinization [19]. Another filaggrin type protein, named filaggrin-2 (FLG2) has already been described in humans since 2009 [20]. Filaggrin 2 has overlapping distribution with filaggrin and is thought to be important for proper keratinization, although much less is known about its effect on cell morphology and nuclear dissolution during keratinization [15,21]. Filaggrin 2 is decreased in humans with AD, particularly in lesional skin, when compared to healthy controls [22]. 

Changes detected on skin biopsies could be due to inflammation. Cytokines modulate the expression of proteins important for skin barrier function [23] such as filaggrin and TJ proteins. Establishment of cell cultures allows study of the differentiation and behavior of keratinocytes minimizing the inflammatory effect. 

Transepithelial electrical resistance (TEER) is a methodology used extensively in humans as a measurement of skin barrier permeability and TJ protein function in vitro when evaluating monolayers of epithelial cells [24,25]. Currently, to the best of the authors’ knowledge, there are neither published reports on TEER in atopic canine keratinocytes nor published observations on atopic keratinocytes behavior in cell culture. Thus, the purposes of this pilot study were: (1) to measure TEER of keratinocytes harvested from biopsies of normal and atopic dogs and grown in culture to create a monolayer; (2) to compare gene and protein expression and pattern of immunofluorescent staining for TJ proteins and filaggrins in keratinocyte cultures.

## 2. Materials and Methods

### 2.1. Animals

All animal procedures were approved by the University of Florida Care and Use Committee (#201910621). All dogs used in this study were research beagles. The atopic dogs (n = 6) were part of a research colony that has been validated as a suitable model of AD [26]. The normal beagles (n = 7) were age matched research beagles with no prior history or evidence of skin disease. 

### 2.2. Biopsy Collection

Biopsies (two 8-mm biopsy punch biopsies) were implemented from the inguinal area in all dogs. In the atopic dogs the skin biopsied was clinically non-lesional. The dogs had not been challenged with allergens to avoid any changes induced by allergen exposure and consequent allergic stimulation. The skin was cleaned with ethanol and betadine before the implementation of the biopsy. Sodium bicarbonate and lidocaine was injected prior to biopsy harvesting. The site was routinely sutured. The biopsy was placed in sterile PBS on ice, then washed with betadine. Each biopsy was cut in half then placed in 1.25 U/L dispase overnight. The epidermis was removed using sterile forceps and floated on TrypLE (Gibco 12563-011) for 30 min.

### 2.3. Cell Culture

Keratinocytes were harvested by agitating the epidermis, then cultured using CellnTech (CnT-09) media. After proliferation these were frozen in cell culture freezing media (Gibco 12638-010) overnight at −80 °C then kept in liquid nitrogen for storage. Cells were seeded onto Lab-TeK II Chamber Slides (ThermoFisher 154526, Waltham, MA, USA) at 1 × 10^5^ cells per well. The time point was labeled as day 0 (D0) once cells reached confluency. 

### 2.4. Transepithelial Electrical Resistance (TEER) Measurement

To measure TEER, 1.875 × 10^4^ cells per well were grown on transwell inserts (costar 3470) in 24-well plates. We used 250 ul CellnTech (CnT-09) media in the well and 500 µL media in the plate below the well. We also seeded 1.1 × 10^5^ cells in wells without inserts, to be able to observe confluency. Confluent readings were taking using the EVOM2 Epithelial Voltohmmeter by World Precision Instruments. The instrument was rinsed in media between each dog and placed in 70% ethanol after all reading were completed each day. TEER was measured daily up to 13 days after confluence.

### 2.5. Real Time Polymerase Chain Reaction (RT-PCR)

RNA was extracted from cells using 5Prime PerfectPure RNA Cell Kit (cat no. FP2302500) following the kit protocol. Reverse transcriptase was performed using Superscript II (Invitrogen 18064-014). Briefly, 1–3 µg RNA was added to a PCR tube with 1.5 µL 25 µM random hexamers (ThermoFisher N8080127) and filled to 17 µL with DEPC H2O. This was heated to 70 °C for 10 min then placed on ice. The following was added to the tube: 6 µL 5X 1st Strand Buffer, 3 µL 0.1 M DTT, 1.5 µL 10 mM dNTP mix (ThermoFisher 10297018), and 1 ul Superscript II. This was heated to 25 °C for 10 min, 42 °C for 45 min, 95 °C for 3 min and kept at 4 °C until removed for use in real time PCR. Real Time-PCR was performed on StepOnePlus Real-Time PCR System (ThermoFisher 4376600) using PowerUp SYBR Green Master Mix (ThermoFisher A25741). Primers were diluted to 50 mM with DEPC H2O, then 10x primer mix was made using 100 µL each forward and reverse primer and 800 µL of DEPC H2O. cDNA was diluted 1:2 before use in RT-PCR. Each well contained 12.5 µL 2X SYBR Green, 2.5 µL 10X primer mix, 2 µL cDNA, 8 µL DEPC H2O. We used the Quantitative ΔΔCt set up on the StepOnePlus Real-Time PCR System. Primers are as follows: 

ZO-1 forward 5-CCCACGAAGTTACGAGCAAGT-3, reverse 5-GGACAACCGCAGCACCAT-3. Claudin1 forward 5-CGAAAAACAACATCTTACCCAACA-3, reverse 5-CACTGGAAGGCGCAGGTT-3. Occludin1 forward 5-ATCCTGCTCGTCCTGAAGAT-3, reverse 5-AGGTGGACTCTCAAAAGGCCTCGATGACAT-3. We used RPL0 as our housekeeping gene.

### 2.6. Protein Extraction

Protein extraction for Western blot analysis was achieved by using RIPA (radioimmunoprecipitation assay) buffer. RIPA buffer and 8M UREA were prepared and HALT protease was added to each well (600 µL RIPA and 250 µL UREA). Media was removed and 400 µL of RIPA protein lysis buffer with protease was added to the well. The bottom of the well was scraped with pipet tip and agitated, avoiding excess bubbles. Content of the well was collected to a clear tube so that pellets could be seen after centrifugation. Additional 200 µL was added to well and collected to same Eppendorf, vortexed and centrifuged at 13,000× *g* for 10 min at 4 °C. Supernatant was removed and 250 µL of 8 M UREA buffer were added and vortexed multiple times to dissolve. Samples were placed in −80 °C until further analysis. 

### 2.7. Western Blot

Protein samples were electrophoresed using Novex^®^ NuPAGE^®^ 4–12% Bis-Tris gel (Product # NP0321BOX), XCell SureLock™ Electrophoresis System (Product # EI0002) and Precision Plus Protein™ WesternC™ Protein Standards (Product # 1610376) at 120 volts for 1 h and 30 min. Proteins were then transferred onto a PVDF Membrane (Product #LC2002) using the XCell II™ Blot Module (Product #EI9051) at 280 mV for 1 h 30 min. The membrane was blocked for one hour with 5% Bovine Serum Albumin (BP1600-100) at 25 °C. The membrane was probed with the relevant primary antibody overnight at 4 °C followed by washes in PBST and HRP conjugate secondary antibody incubation (Amersham ECL Western Blotting Detection Kit RPN2108, 1:2000 dilution) for 1 h at 25 °C. Chemiluminescent detection was performed using Amersham ECL Western Blotting Detection Kit RPN2108.

### 2.8. Immunofluorescence (IF) Staining

Cells were fixed using 3.7% PFA on either D0 or D6 then rinsed in PBS and diH2O. Slides were dried overnight at RT and 20 min at 60 °C. All dilutions for immunofluorescence were diluted with common antibody diluent (BioGenex HK156-5K). Antigens were retrieved by placing slides in Tris-EDTA pH 9 buffer for 15 min in a rice cooker (Aroma ARC-150SB) on ‘steam’, then cooled in rice cooker for 10 min and washed with PBS. The slides were incubated with Power Block (Biogenex HK083-50K) for 10 min, power block was tapped off without being rinsed and blocked again using normal donkey serum (Jackson ImmunoResearch 017-000-121) diluted to 10% for 20 min. Slides were washed with PBS and incubated with primary antibody overnight at 4 °C. Slides were washed with PBS. Secondary donkey anti mouse IgG 488 (ThermoFisher A10042) and donkey anti rabbit 594 (ThermoFisher A21202), diluted 1:500, was added and incubated for 45 min in the dark RT. Slides were washed with PBS, coverslipped with VECTASHIELD Antifade Mounting Medium with DAPI (H-1200), and sealed using nail polish. Antibodies used were ZO-1 (ThermoFisher 339100) 1:200; Occludin (ThermoFisher 331500) 1:300; Claudin 1 (abcam ab15098) 1:20; Mouse IgG1 Isotype Control (ThermoFisher MA5-14453) 1:300; Filaggrin (courtesy of Baylor College of Medicine, rabbit polyclonal) 1:150; and Filaggrin 2 (courtesy of Dr. Santoro, rabbit polyclonal) 1:150.

### 2.9. Imaging and Data Analysis

Fluorescent pictures were taken at 20 X on an EVOS fluorescence microscope. Intensity and shutter speed were determined by testing settings on both atopic and normal chambers. The same settings were used for each antibody. Five pictures of each antibody for each chamber were taken to get a good representation of all cells in the chamber. Pictures were scored subjectively by four observers. For the TJ proteins (occludin, claudin, and ZO-1) observers scored for continuity (0–3). For the filaggrin proteins, observers scored 3 attributes: intensity (0–5), location (nucleus or cytoplasm), and whether the staining was discrete (yes/no). A set of pictures from each subject was also scored for cell size (0–3) and if there were multiple cell sizes (yes/no). 

### 2.10. Confocal Microscopy

Confocal microscopy is commonly used for imaging of TJ [27]. 

In this study, confocal microscopy was performed on Nikon A1RMPsi-STORM4.0 on the 60 X water objective. Settings for each confocal image were optimized by testing settings on one atopic and one normal slide. These settings were optimized for each antibody, then kept the same through the rest of the microscopy. One z-stack was captured for each set of antibodies for each dog. This z-stack was opened in NIS Elements and transformed into a 3D movie, then exported to an mp4 file. Videos were used for descriptive purposes and no objective analysis was carried out on them.

### 2.11. Data Analysis and Statistics

#### 2.11.1. TEER

Data from the EVOM2 Epithelial Voltohmmeter is present in Ohms/cm^2^. The atopic vs normal dogs were compared over time using a two-way ANOVA. This was performed in Graph Pad Prism 8.

#### 2.11.2. RT-PCR

The real time PCR data was analyzed as follows. We subtracted the RPL0 Ct values from the sample Ct values to get the ΔCt. The ΔCt for day 0 was subtracted from ΔCt for day 6 to get the ΔΔCt. The fold change was calculated by using 2^−ΔΔCt^. Statistical analysis was done on log 2 value of 2^−ΔΔCt^. All graphs show value 2^−ΔΔCt^ the fold change, with the *y* axis on a log scale. Note the Y axes on the graphs are different scales. Unpaired two-tailed *t*-tests were performed for: fold change from D0 to D6, atopic vs normal; atopic D0 vs normal D0; atopic D6 vs normal D6; atopic D0 vs atopic D6; and normal D0 vs normal D6. These statistical tests were performed in Graph Pad Prism 8. Correlations between gene expression (ΔCt values) and TEER values was analyzed using Pearson product moment correlation (SAS software, Cary, NC, USA). 

#### 2.11.3. Immunofluorescent Staining Picture Analysis, Videos and Western Blot 

The score for each picture was averaged between the four observers and the five images if the variable was an integer. If the variable was categorical, the percent of observers who indicated each category was calculated for each picture. If the parameter was a yes/no question, the sum of the ‘yes’ was taken for each picture, then the sum of the ‘yes’ for each chamber and dog (0–20 score) was taken and this score was used. The Shapiro–Wilk test was used to determine normality. If these data were normally distributed a two-way repeated measures ANOVA and multiple comparisons were performed using the two-stage linear step-up procedure of Benjamini, Krieger, and Yekutieli. If these data were not normally distributed a series of Wilcoxon matched rank tests (to compare days) and Mann–Whitney U tests (to compare groups) were performed. 

Subjective scores for immunofluorescence staining were compared in Graph Pad Prism 8 using a two-way ANOVA. ANOVA was performed to compare size of keratinocytes between normal and atopics. Effect of time, subject and group were considered. Videos prepared from the images taken using the confocal microscope were used for observational purposes only and no objective measurements or quantification was applied. Paired T tests were done to analyze differences in Western blots.

## 3. Results

### 3.1. TEER

**Figure 1:**
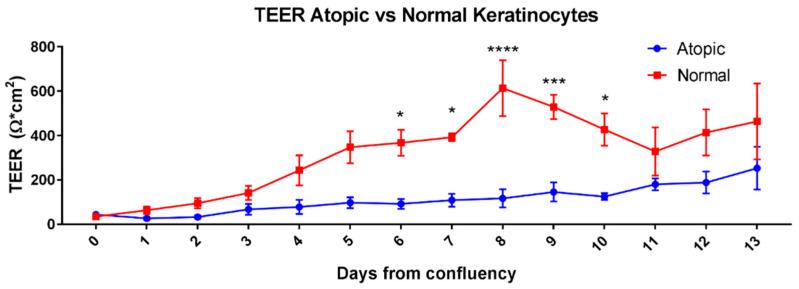
Means and SEM of TEER values of normal and atopic keratinocytes grown in cell culture starting on the first day of confluence (D0) and for 13 days afterwards. * indicates *p* values < 0.05; *** *p* ≤ 0.01 and **** *p* < 0.0001).

TEER of normal keratinocytes grown in monolayer had values that were significantly higher than atopic keratinocytes. A two-way repeated measures ANOVA showed a significant effect of time (*p* < 0.0001), group (*p* < 0.0001) and group x time interaction (*p* < 0.0001). The difference between two groups reached a peak at D8 (Figure 1). Sidak multiple comparison test showed that the difference started to be significant at D6 (*p* = 0.0373) and remained significant until D10 (*p* = 0.028 at D7, *p* < 0.0001 at D8, *p* = 0.0006 at D9, and *p* = 0.01 at D10). 

### 3.2. RT-PCR

**Figure 2:**
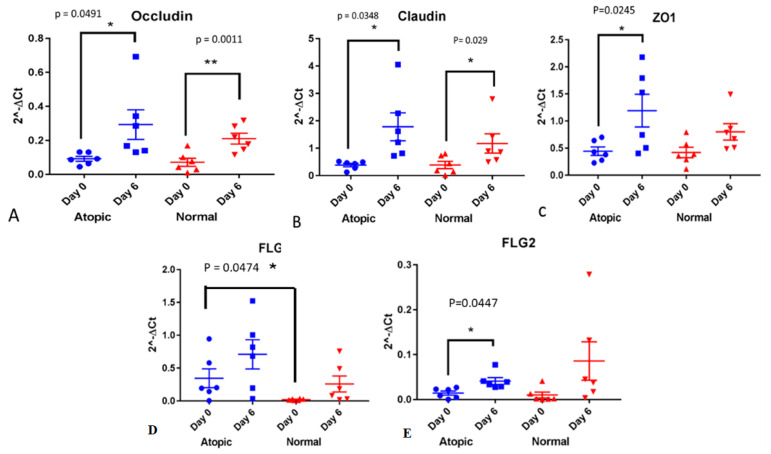
Gene expression of Tight Junction proteins and filaggrins on first day of confluence, day 0 (DO) and day 6 (D6). All numbers and statistical analyses are presented as 2^(-deltaCt). Note that the *y* axes on these graphs use different scales (**A**–**E**). * indicates *p* values that are *p* < 0.05 and ** is for *p* < 0.005.

Gene expression of occludin was not different between atopics and normals on both D0 and D6. In each group, it significantly increased on D6 compared to D0 (*p* = 0.0491 for atopics and *p* = 0.0011 for normals (Figure 2A). Gene expression of claudin was not different between normals and atopics on either day and significantly increased in both groups on D6 compared to D0 (*p* = 0.034 for atopics and *p* = 0.0296 for normals, Figure 2B). Gene expression of ZO-1 was not different between normals and atopics and significantly increased only in the atopic group (*p* = 0.0245, Figure 2C). Gene expression of filaggrin was not statistically significant between groups and overtime while the one of filaggrin 2 was significantly increased in the atopic keratinocytes (*p* = 0.0447). 

**Figure 3:**
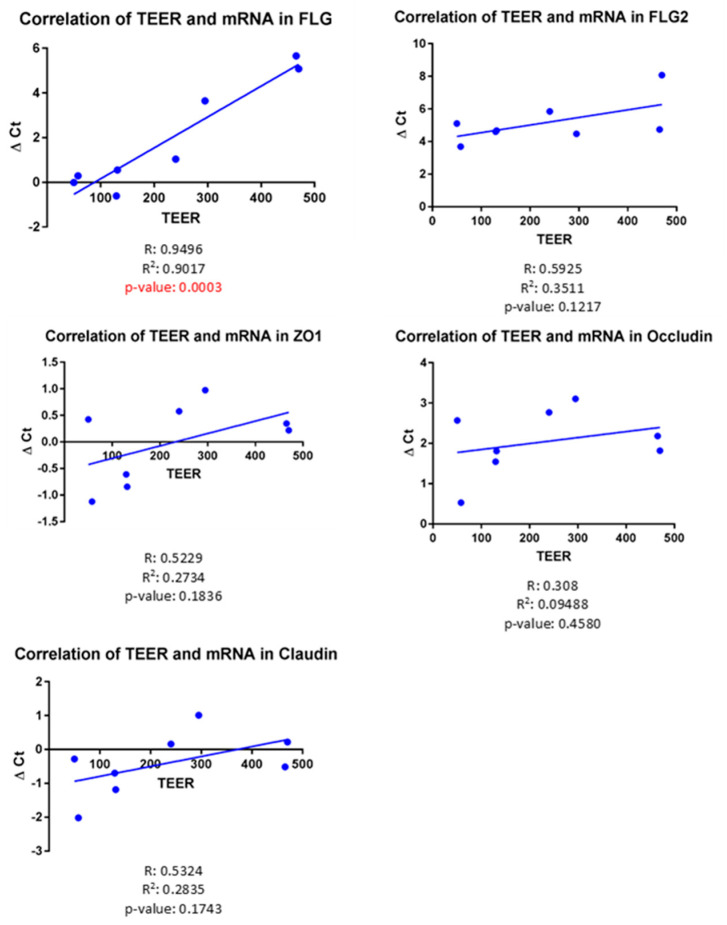
Correlation between TEER and the gene expression of filaggrins and TJ proteins on D6. The only statistically significant correlation was found between gene expression of filaggrin and TEER (r = 0.95, *p* = 0.0003), combining both normal and atopic samples.

TEER and gene expression of filaggrin were significantly correlated in a positive way (r = 0.95, *p* = 0.0003, Figure 3). No significant correlation was found between any of the TJ proteins and TEER on D6, combining both normal and atopic samples. 

### 3.3. Subjective Evaluation of IF Images 

#### 3.3.1. Tight Junction Proteins

**Figure 4:**
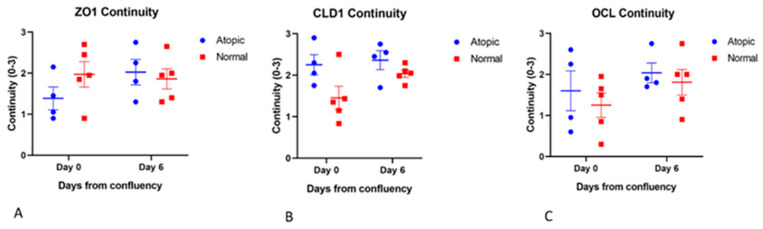
Average of subjective scoring done by four observers on five pictures of each antibody for each chamber (**A**–**C**). Observers were not aware of the source of the cells scored. No significant differences were found for any of the TJ proteins between groups or overtime in terms of continuity. For this scoring, four atopic cell cultures and 5 normal cells cultures were used.

No difference for continuity was found for occludin, claudin 1, and ZO-1 between normals and atopics and between D0 and D6 (Figure 4). 

#### 3.3.2. Filaggrins

**Figure 5:**
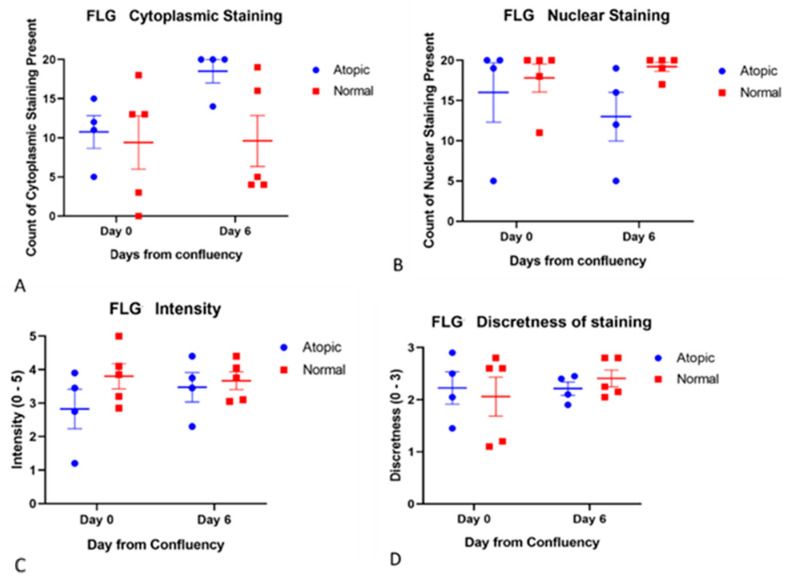
Filaggrin staining was scored for location, intensity, and discreteness by four observers unaware of the source of the cells (**A**–**D**). No statistically significant differences were found between groups and from D0 to D6 for any of these parameters. For this scoring, four atopic cell cultures and five normal cell cultures were used.

**Figure 6:**
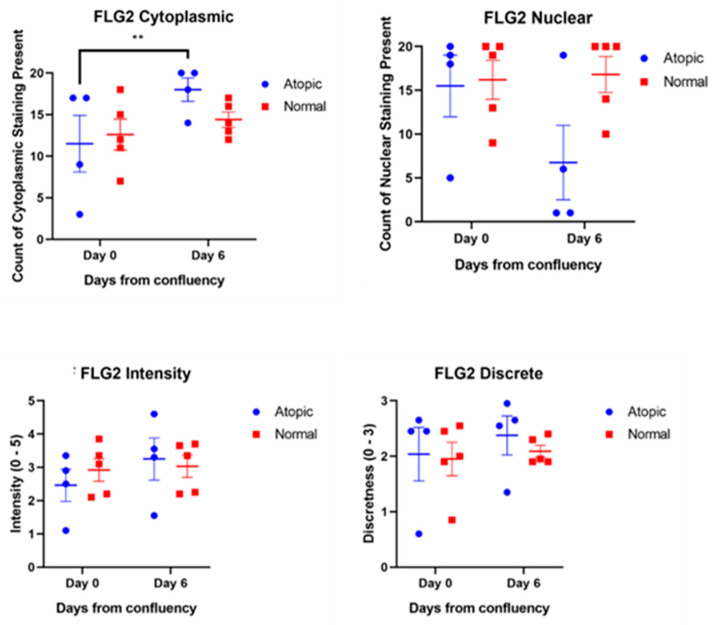
Filaggrin 2 staining was scored for location, intensity, and discreteness by four observers unaware of the source of the cells. The increase of cytoplasmic filaggrin-2 on D6 was significant in atopics (*p* = 0.0067). The decrease of staining of filaggrin-2 in the nucleus at D6 approached significance in the atopics (*p* = 0.055). For this scoring four atopic cell cultures and five normal cell cultures were used. ** is for *p* < 0.005.

Filaggrins staining was detected both in the nucleus and in the cytoplasm. Filaggrin location was not significantly different over time (Figure 5). Filaggrin 2 location in the cytoplasm significantly increased from D0 to D6 in atopic keratinocytes (Figure 6). 

No statistically significant difference in intensity of the staining for either filaggrin was found with subjective evaluation of the images by the observers. 

#### 3.3.3. Cell Size

**Figure 7:**
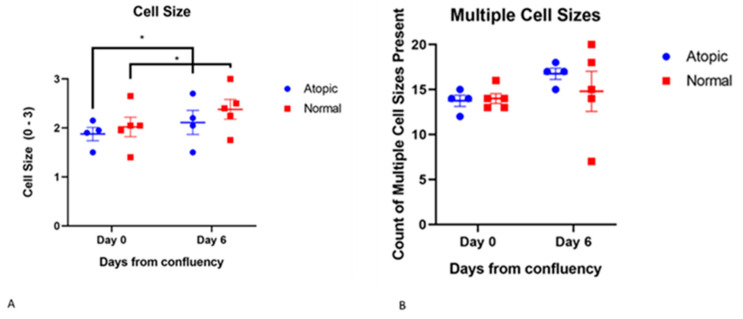
Size significantly increased from D0 to D6 in both groups (effect of time, *p* = 0.0076) but there were no differences between groups (**A**,**B**). No differences were found in terms of frequency of multiple cell sizes between normal and atopics. For this scoring, four atopic cell cultures and five normal cell cultures were used. * indicates *p* values that are *p* < 0.05.

ANOVA showed increased cell size from D0 to D6 in both normal and atopic keratinocytes (effect of time, *p* = 0.0076, Figure 7) but no differences existed between groups. Significant effect of subject (*p* = 0.0022) was found indicating that cell size was more related to the individual rather than whether it was normal or atopic. 

### 3.4. Western Blot

**Figure 8:**
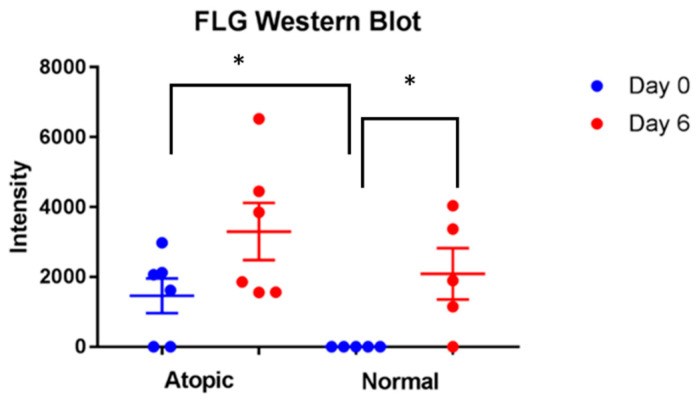
Significant increase of filaggrin was found in normal keratinocytes on D6 compared to D0 (*p*= 0.046). Atopic keratinocytes had significantly higher amounts of filaggrin compared to normals on D0 (*p* = 0.032). * indicates *p* < 0.05. For this analysis, five normal and six atopic cell cultures were used.

Significant increase of filaggrin was found in normal keratinocytes on D6 compared to D0 (*p* = 0.046, Figure 8). Atopic keratinocytes had significantly higher amounts of filaggrin compared to normals on D0 (*p* = 0.032, Figure 8). No significant difference for filaggrin amounts were found on D6 between normal and atopic keratinocytes.

**Figure 9:**
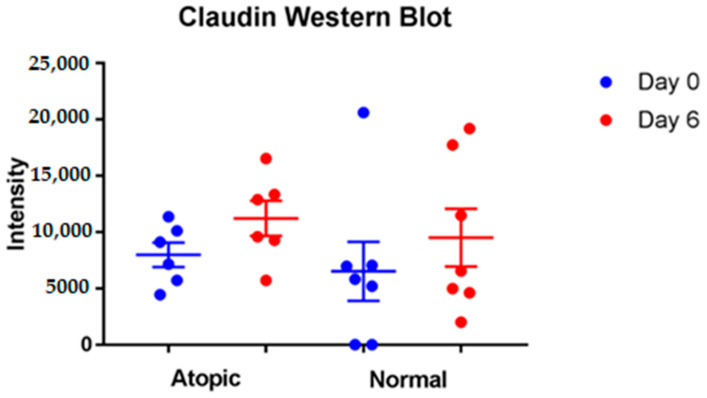
No significant differences for Claudin 1 were found overtime and between normal and atopic keratinocytes. For this analysis, seven normal and six atopic cell cultures were used.

No significant differences for Claudin were found between normal and atopic keratinocytes and between D0 and D6 (Figure 9).

### 3.5. Images and Threedismensional Videos Made Using Confocal Miscroscope

**Figure 10:**
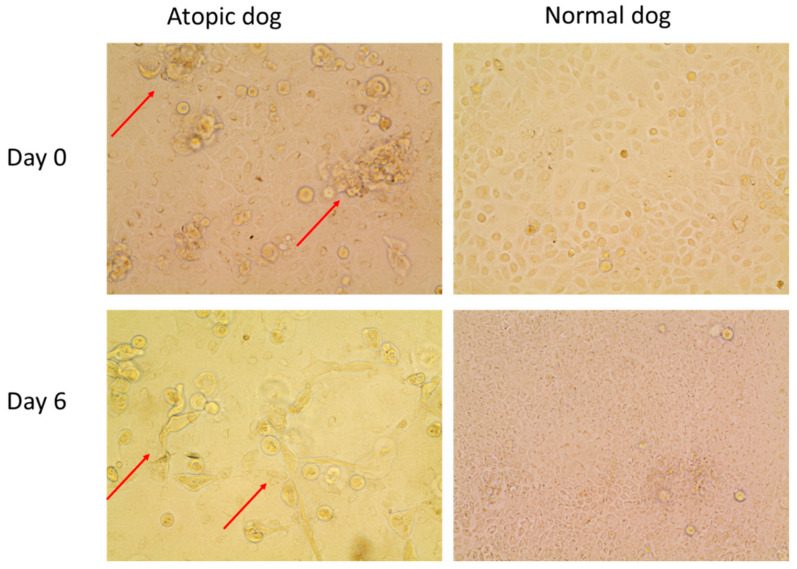
Pictures of unstained cell cultures of normal and atopic dogs at day 0 (first day of confluence) and day 6. Note how the atopic cells create little irregular accumulations of cells (“domes”) as indicated by the red arrows, while the normal cells are more evenly distributed, creating a flat monolayer.

**Figure 11:**
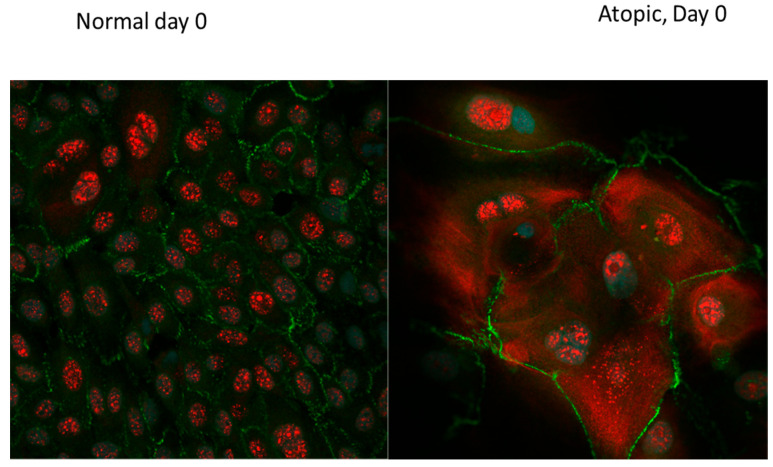
Confocal microscopy images of normal and atopic keratinocytes at the first day of confluence (D0). Confocal microscopy was performed on Nikon A1RMPsi-STORM4.0 on the 60 X water objective. On the left, keratinocytes harvested from a normal dog while on the right, keratinocytes harvested from an atopic dog. On D0, atopic keratinocytes appeared larger, more differentiated and with more variation in size compared to the normal keratinocytes which appeared smaller and more uniform in size. Filaggrin (stained in red) was evident only in the nucleus in the normal keratinocytes, while in the atopic keratinocytes, it was present both in the nucleus and in the cytoplasm. This distribution was consistent with a further stage of differentiation. ZO-1 (stained in green) was evident on the membrane of the keratinocytes in both normal and atopic keratinocytes. Light blue was DAPI, a fluorescent DNA stain, to locate the nucleus of the cells.

On D0, normal keratinocytes (Figure 10 and Figure 11, Video S1 in Supplementary Material), were organized in a flat monolayer. 

Filaggrin (stained in red) was evident as discrete filaments in the nucleus of normal keratinocytes. On the same day, atopic keratinocytes (Figure 11, Video S2 in Supplementary Material) appeared larger with filaggrin present both in the nucleus and in the cytoplasm. The cytoplasmic location was consistent with a more advances stage of differentiation. The appearance of filaggrin in the cytoplasm of atopic cells appeared finer and less discrete than in the nucleus. ZO-1 (stained in green) was evident on the membrane of the keratinocytes. 

**Figure 12:**
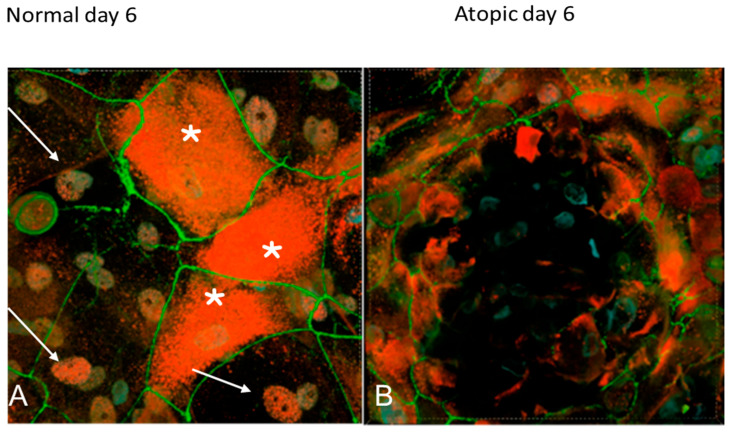
Confocal microscopy images of normal and atopic keratinocytes six days after confluence (D6). In normal keratinocytes (**A**), filaggrin (stained in red) is visible in the cytoplasm in some cells (white asterisks) and in the nucleus in others (white arrows). Atopic keratinocytes (**B**) have created little dome structures. ZO-1 (stained in green) was evident on the membrane in both normal and atopic keratinocytes. Light blue was DAPI, a fluorescent DNA stain, to locate the nucleus of the cells.

On D6, filaggrin was evident primarily in the cytoplasm of both normal and atopic keratinocytes (Figure 12A). Atopic keratinocytes had grown to create large, dome-like structures (Figure 12B).

**Figure 13:**
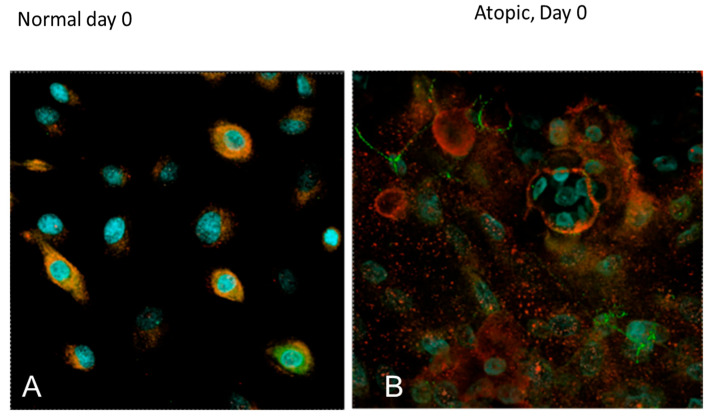
Confocal microscopy images of normal and atopic keratinocytes at the first day of confluence (D0). Confocal microscopy was performed on Nikon A1RMPsi-STORM4.0 on the 60 X water objective. On the left (**A**), keratinocytes harvested from a normal dog while on the right (**B**), keratinocytes harvested from an atopic dog. Filaggrin 2 is stained in red and occludin is in green. Filaggrin 2 shows primarily in the cytoplasm in these images. Light blue was DAPI, a fluorescent DNA stain, to locate the nucleus of the cells.

On D0, filaggrin 2 staining was present in the cytoplasm of both normal and atopic keratinocytes (Figure 13). In normal keratinocytes, (Figure 12A) filaggrin 2 appeared as discrete filaments (Video S3, in Supplementary Material), while in the atopic keratinocytes it appeared disorganized and had a combination of discrete and finer filaments (Video S4, in Supplementary Material). 

**Figure 14:**
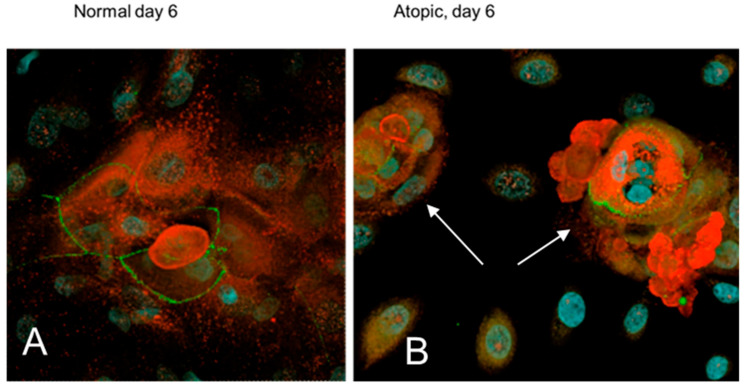
Filaggrin 2 staining (red) in normal (**A**) and atopic (**B**) keratinocytes on D6. Confocal microscopy was performed on Nikon A1RMPsi-STORM4.0 on the 60 X water objective. Atopic keratinocytes (**B**) have formed isolated round accumulation of cells into dome-like structures (white arrows). Green staining shows distribution of occluding and blue staining (DAPI) shows the nucleus.

On D6, filaggrin 2 was visible in the cytoplasm of both normal and atopic keratinocytes (Figure 14).

## 4. Discussion

In our pilot observations, the growth behavior of keratinocytes harvested from non-lesional canine atopic skin was different from that of keratinocytes harvested from normal dogs. Atopic keratinocytes grew upward, first creating dome-like structures and only later moved laterally to form a monolayer. For this reason, on the first day of confluence, atopic keratinocytes were more advanced in their differentiation compared to the normal keratinocytes. Advanced keratinization in atopic keratinocytes was demonstrated by the detection of filaggrin filaments both in the nucleus and cytoplasm. Normal keratinocytes on D0 showed filaggrin only in the nucleus, which is an earlier stage in the processing of filaggrin. Translocation of filaggrin from the nucleus to the cytoplasm is part of the normal differentiation of keratinocytes [28,29]. In our study, normal keratinocytes grew in a more uniform and organized fashion to form a flat monolayer. We also found that TEER was different between the normal and the atopic keratinocytes, with atopics having significantly lower TEER compared to the normal keratinocytes. In our study, TEER positively correlated with filaggrin gene expression. 

To the best of the authors’ knowledge, studies of canine atopic keratinocytes in cell culture are limited and have been done primarily by our group [30]. Other studies published in the veterinary literature have typically used cell lines like the canine epidermal keratinocyte progenitors (CPEK) [31,32] or canine keratinocytes harvested from normal dogs [33]. Also, to the best of the authors’ knowledge, this is the first report in veterinary medicine on the application of TEER in canine atopic dermatitis. Another study on TEER was done by our group comparing normal keratinocytes and CPEKs [34]. 

The measurement of TEER is used to assess permeability of epithelia in vitro. The typical approach is to assess TEER using an established cell culture and then test the effect of various factors [35,36,37]. Our study was different, as we made a direct comparison between primary normal and atopic keratinocyte cultures. The decreased TEER observed in the atopic keratinocyte culture (consistent with increased epithelial permeability) could possibly be attributed to the growth pattern of the keratinocytes rather than to the expression of TJ proteins. Although TEER was not measured until the cells were confluent, the uneven pattern of growth of the atopic keratinocytes, with the creation of irregular domes rather than a flat organized monolayer (like the normal cells did), most likely played a role in the TEER results. The authors could not find another study in the human literature that had described the behavior of human atopic keratinocytes grown in cell culture and whether the creation of these dome-like structures is a canine feature or something that atopic keratinocytes do across species. The authors have grown atopic keratinocytes on numerous other occasions and this behavior was found to be a consistent feature in atopic cells. The visualization of the cell cultures in 3D videos was useful for observation of these domes.

The significant positive correlation we found between filaggrin gene expression and TEER is not surprising, as filaggrin is important for barrier function. A study on epithelial esophageal permeability showed that TEER worsened when filaggrin decreased [17]. The correlation between the TJ proteins and TEER warrants additional investigation as our study had a small sample size. It is conceivable that a larger number of samples may provide more information about correlations between TEER and TJ proteins in dogs.

Our study is to be considered a pilot study due to the low number of dogs used for the cultures, yet it provides an interesting insight in the growth behavior of atopic keratinocytes. In our study, keratinocytes were used at second or third passage to minimize the contamination of other cells. Nevertheless, it is possible that the influence of the cytokine milieu of origin still persisted in cell culture and that this lingering effect modulated the keratinocyte behavior observed in the atopic cells. Th2 cytokines have been shown to stimulate keratinocyte proliferation in vitro [38]. It is also possible that intrinsic differences in cytokine production with paracrine effects may exist between atopic and normal canine keratinocytes. From studies on human keratinocytes, it is known that Granulocyte-Macrophage Colony Stimulating Factor (GM-CSF) is produced by keratinocytes shortly after injury [39] and mediates epidermal proliferation in an autocrine manner [40]. Higher constitutive GM-CSF gene expression has been reported in keratinocyte cultures established from non-lesional skin of human atopic patients [41,42]. The larger amounts of GM-CSF produced by human atopic keratinocytes are considered to play a role in the establishment and in the perpetuation of disease. More specifically, the increased production of GM-CSF by human keratinocytes is considered to play a role in the increased survival of monocytes and granulocytes and excessive proliferation of the keratinocytes themselves [43]. 

On the veterinary side, GM-CSF production has been studied in CPEKs and it was increased by stimulation with allergens such as house dust mites [44], and suppressed by cytokines like gamma interferon [45]. It is currently unknown whether canine atopic keratinocytes constitutively overexpress GM-CSF and, if so, whether this could explain some of our findings. 

Epidermal Growth Factor (EGF) is important for keratinocyte motility [46,47,48]. Epidermal Growth Factor is important for skin development and homeostasis [49,50], and EGF administration has been shown to suppress inflammation in mouse models of atopic dermatitis [51]. Epidermal growth factor receptor transcriptional levels are lower in lesional AD skin as compared to normal healthy skin [52]. Dysregulation in EGF receptor activation has been linked to GM-CSF expression in humans, both in vivo and in vitro [53]. It is possible that such dysregulation may also exist in canine atopic dermatitis and could explain the decreased lateral motility of atopic keratinocytes in culture and the different growth behavior. Future studies should evaluate the effect of various factors on the proliferation and migration of canine keratinocytes in cell culture and the expression of EGFR in atopic keratinocytes. 

In our PCR studies, the statistically significant increase of gene expression of TJ proteins and filaggrins in atopic keratinocytes over time is consistent with differentiation. The increase of TJ proteins like occludin and claudin was also significant over time for the normal keratinocytes but no differences were found between normal and atopics at any time point. In the evaluation of immunofluorescent staining for TJ proteins we found no difference between normals and atopics for continuity. This finding is different from previous reports of immunohistochemical staining, which reported decreased expression in atopics [15,54] as well as a patchier pattern (at least for ZO-1, [15]). It is possible that the decreased expression of TJ proteins reported in immunohistochemical studies between normal and atopic samples could have been secondary to inflammation rather than to a primary issue. Moving forward, it would be of interest to grow normal keratinocytes and expose them to various inflammatory cytokines and measure the expression of TJ proteins. This has been done in humans with a variety of epithelial cells and it is known that Th2 cytokines suppress TJ protein expression [12,55,56]. 

Our study is the first one that reports the staining pattern of both filaggrin and filaggrin 2 in normal and atopic keratinocytes in cell culture. Previous reports on filaggrin staining in the veterinary literature have been confusing, as some authors published studies on what is now known to be filaggrin 2 [57] while others actually stained filaggrin [58]. In those early publications the authors simply refer to “filaggrin”. Our study aimed to stain both filaggrins and to observe possible differences. What we observed is that, as reported in humans, the two filaggrins have similar patterns of distribution [20,21]. Currently, little is understood about the specific differences between filaggrin and filaggrin 2. They are both believed to play important and somewhat similar roles in barrier function [59]. Interestingly, the consequences of deficiencies are slightly different [60]. For example, in filaggrin-2-knockdown mice an increase in pH of the skin occurs, while in filaggrin-knockdown animals there is no change in pH [60]. Which filaggrin is of most relevance in canine atopic dermatitis is not known. The pH of canine atopic skin is higher than in normal dogs [61], so it could be speculated that maybe filaggrin 2 is more important in the canine disease, although it is most likely that the change in pH is multifactorial.

In our subjective evaluation of the images by observers unaware of the source of the cells evaluated in the images, no differences were reported between normals and atopics in terms of intensity of staining for filaggrins between normals and atopics. It is possible that the decreased staining reported in skin biopsies of atopic dogs [62] may be due to inflammation rather than to a primary issue. Atopic skin shows a low level of inflammation even when it is clinically non-lesional as it was in the case of the biopsy sites used to harvest the keratinocytes for our study. Additionally, it is possible that the lack of differences found in our study are due to the small sample size and that, with a larger sample size, differences may become more evident. Differences in filaggrin synthesis and metabolism [4,26,63] between normal and atopic dogs have been proposed but it is unclear whether they are a primary atopic trait versus one secondary to inflammation. Currently, filaggrin mutations do not appear to be a well-documented risk factor in dogs, and breed and geographical variations exist [4,64]. 

## 5. Conclusions

To conclude, in our pilot observations, interesting differences exist in the growth behavior in cell culture between atopic and normal keratinocytes. Atopic keratinocytes created dome-like structures rather an organized flat monolayer like normal cells did. The reasons for this different growth behavior are unknown at this time. No obvious differences in TJ protein and filaggrin expression were evident between normal and atopic keratinocytes, suggesting that previously reported differences in intensity of staining for both filaggrin and TJ protein in skin biopsies might have been caused by inflammation rather than being a primary atopic trait. Filaggrin and filaggrin 2 appear to have similar distribution.

## Supplementary Materials

The following supporting information can be downloaded at: https://www.mdpi.com/article/10.3390/vetsci9070329/s1, Videos S1 and Videos S2. Videos showing a rotating tridimensional view of the monolayer of normal and atopic keratinocytes on the first day of confluence (D0). While normal keratinocytes created a flat monolayer of uniform looking keratinocytes, atopic keratinocytes formed raised dome-like structures of more irregular looking cells. Videos S3 and S4. Double click to play the videos showing a rotating tridimensional view of the normal and atopic keratinocytes on D0 stained for filaggrin 2. In normal keratinocytes (Video S3), filaggrin 2 (stained in red) appeared as discrete filaments, while in the atopic keratinocytes (Video S4) appeared disorganized and had a combination of discrete and finer filaments. Green staining shows occludin.
